# Total knee arthroplasty conversion after a failed lateral closing wedge high tibial osteotomy with knee hyperextension and secondary ankle degeneration: A case report

**DOI:** 10.1097/MD.0000000000007473

**Published:** 2017-07-21

**Authors:** Chen Yao, Xingquan Xu, Sheng Zhou, Xiaoxiao Song, Dongquan Shi, Qing Jiang

**Affiliations:** a Department of Sports Medicine and Adult Reconstructive Surgery, Nanjing Drum Tower Hospital, Clinical College of Nanjing Medical University; b Department of Sports Medicine and Adult Reconstructive Surgery, Nanjing Drum Tower Hospital Affiliated with the Medical School of Nanjing University; c Laboratory for Bone and Joint Diseases, Model Animal Research Center, Nanjing University, Nanjing, Jiangsu, China.

**Keywords:** conversion, high tibial osteotomy, lateral closing wedge, total knee arthroplasty

## Abstract

**Rationale:**

High tibial osteotomy (HTO) has been used widely for medial compartment knee osteoarthritis to correct the deformity and relieve symptoms, especially in young patients who are willing to maintain the high activity level. However, the change of bone morphology, ligament imbalance, limb malalignment, and other complications may influence the short-term outcomes of HTO. Some cases may even require conversion to TKA shortly after HTO because of the loss of correction or pain due to accelerated osteoarthritis.

**Patient concerns:**

A 43-year-old female patient presented with persistent pain of both the left knee and the ankle. She underwent a lateral closing wedge HTO two years ago. Radiographies showed The Kellgren-Lawrence (K-L) grade IV osteoarthritic change and hyperextension (HE) of the left knee and the degeneration of the left ankle.

**Diagnoses:**

A failed lateral closing wedge high tibial osteotomy with knee hyperextension and secondary ankle degeneration.

**Intervention:**

A posterior-stabilized TKA conversion and postoperative rehabilitation were performed.

**Outcomes:**

The operation corrected the HE deformity and relieved the pain at the level of the left knee. However, the secondary change of the left ankle was irreversible.

**Lessons:**

A failed lateral closing wedge HTO might speed up the degeneration of the knee and increase extra technical issues in the following TKA. What is more, the secondary osteoarthritis and deformity of the ankle cannot be ignored.

## 1 Introduction

When HTO was introduced for the first time in 1960s,^[1]^ it has been regarded as an ideal treatment strategy for medial compartment knee osteoarthritis. By shifting the weight-bearing loading from the impaired medial compartment to the relatively unaffected lateral compartment, HTO can correct the varus deformity and relieve persistent pain of the knee. HTO is used widely to delay the need for TKA, especially among young or active patients with varus osteoarthritis and patients could retain high activity level post-surgery.^[2]^ HTO could provide favorable clinical outcomes in up to 80% patients with appropriate patient selection and careful operative techniques.^[3,4]^ However, it was also reported that over 20% patients undergoing HTO may need conversion to TKA due to the progressing osteoarthritis and the major of the failures result from errors in surgical techniques.^[5]^

Medial opening wedge HTO and lateral closing wedge HTO are the 2 most commonly performed HTO techniques.^[6]^ They have respective characteristics such as the change of the tibial slope, patellar height, and ligament tension.^[7]^ Compared to medial opening wedge HTO, lateral closing wedge HTO allows early weight bearing with the large contact surface of cancellous bone and the fast bone union at the osteotomy site. However, this procedure will shorten the lower limb and disrupt proximal tibiofibular joint, with the risk of neurovascular injury and compartment syndrome.^[8]^ What is more, the removal of the proximal tibial bone stock after lateral closing wedge HTO may increase the technical issues during the following TKA.^[9]^ In this case report, we described a TKA conversion in a patient who had a failed lateral closing wedge HTO. The secondary changes of knee and ankle and the surgical issues of the TKA following the failed lateral closing wedge HTO were also analyzed.

## 2 Case presentation

**Figure 1:**
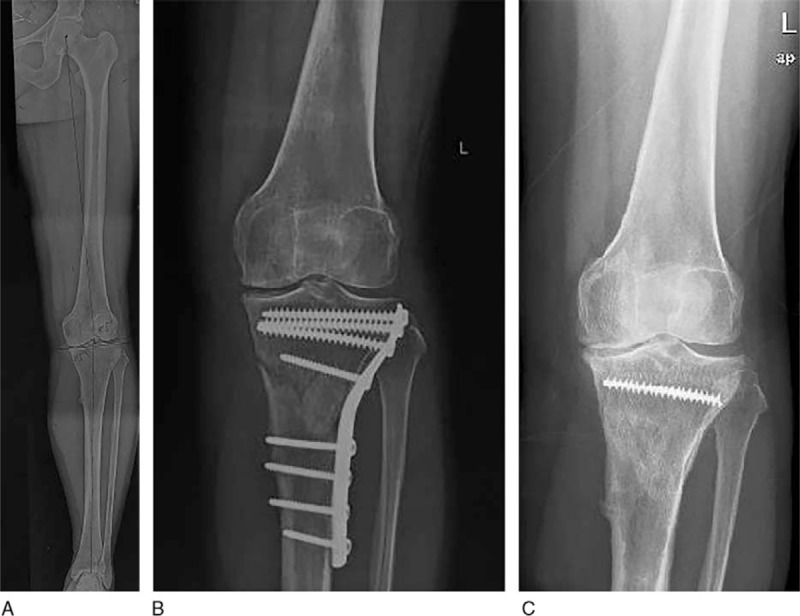
The preoperative x-ray films of the left knee. (a) The x-ray of the left leg before HTO showed medial compartment knee osteoarthritis. (b) The x-ray of the left knee immediately after the HTO 3 years ago. (c) The K-L grade IV osteoarthritic change of the left knee was documented. The fixation had been mostly removed except a snapped screw left in the proximal part of the tibia. HTO = high tibial osteotomy, K-L = Kellgren–Lawrence.

A 43-year-old female patient, who complained serious pain of the left knee and ankle for about 3 years, presented to our clinic in October 2016. She underwent a lateral closing wedge HTO for the medial compartment knee osteoarthritis in an outside institution 3 years prior to this visit (Fig. 1A and B). She had to limp after the surgery owing to the left limb shortening and suffered persistent pain of left knee and ankle. Physical examination showed a mild varus of the left knee. The left knee can actively flex to 120° and had a prominent hyperextension (HE) of about 10°. The Hospital for special surgery (HSS) score of the left knee was 69. In addition, the left ankle was swelling and showed abnormal plantar flextion.

**Figure 2:**
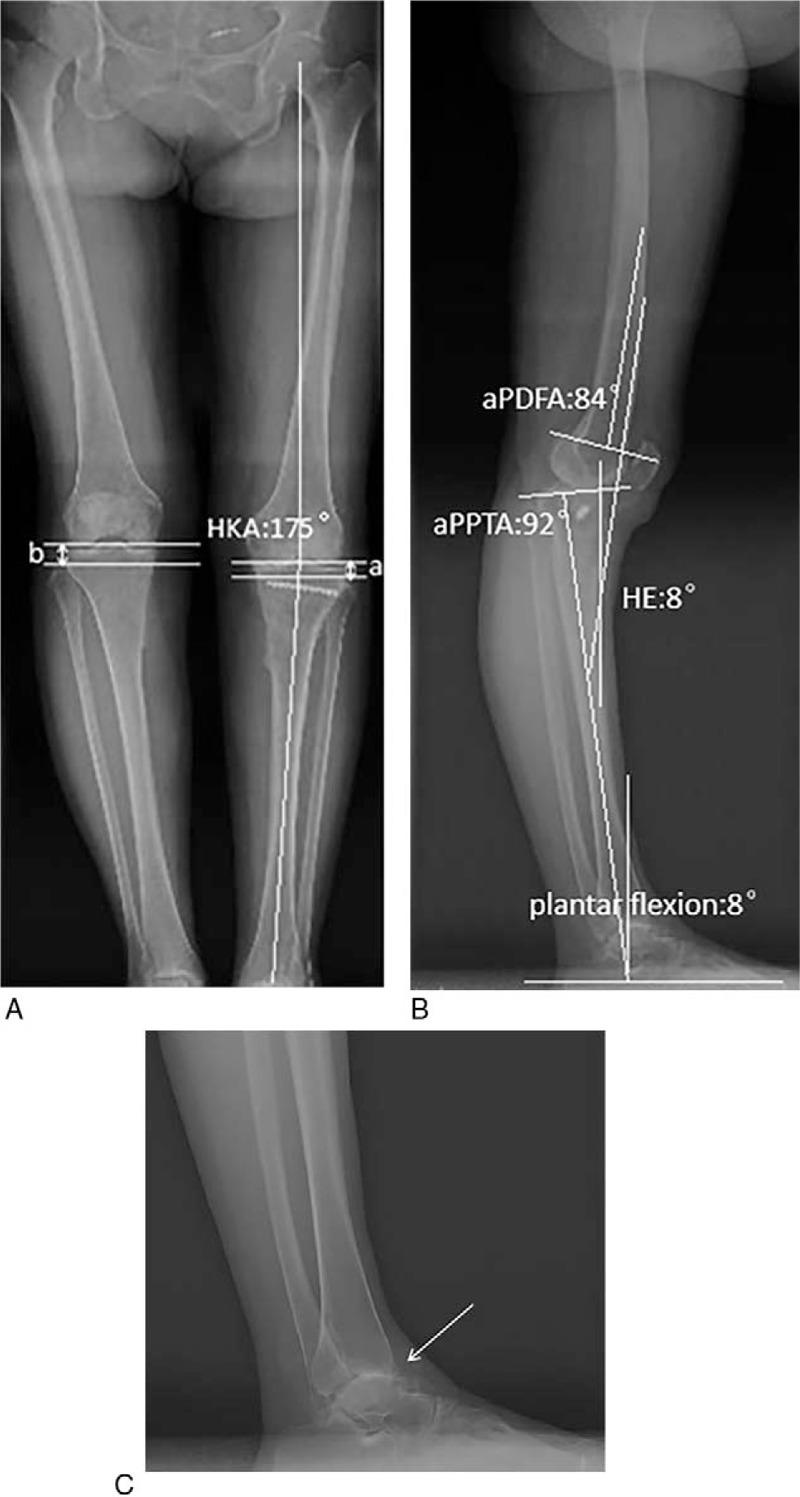
The preoperative weight-bearing x-ray films. (a) The left HKA angle was 175°. The left JLH (a) measured from the fibular head was lower than the right one (b) and the oblique pelvis compensated for the shortening of the left limb. (b) This patient had 8°HE and moderate posterior subluxation. The aPDFA was 84° and the aPPTA was 92°. The tibial recurvatum deformity mainly accounted for the HE and the 8°plantar flexion compensated for it. (c) The secondary osteoarthritis of the ankle were observed (the white arrow). aPDFA = anatomic posterior distal femoral angle, aPPTA = anatomic posterior proximal tibial angle, HE = hyperextension, HKA = hip-knee-ankle, JLH = joint line height.

The Kellgren-Lawrence (K-L) grade IV osteoarthritic change of the left knee was documented (Fig. 1C). The fixation had been mostly removed except a snapped screw left in the proximal part of the tibia. The weight-bearing x-ray (Fig. 2A) showed the recurrence of the varus deformity. The hip-knee-ankle (HKA) angle was 175°. The left joint line height (JLH) measured from the fibular head was lower than the right one and the oblique pelvis compensated for the shortening of the left limb (Fig. 2B). The left knee had 8°HE and moderate posterior subluxation on the sagittal view (Fig. 2C). The anatomic posterior distal femoral angle (aPDFA) was 84°. The anatomic posterior proximal tibial angle (aPPTA) was 92°, which suggested that the tibial posterior slope was decreased. According to the knee malorientation test on the sagittal plane introduced by Paley,^[10]^ the tibial recurvatum deformity mainly accounted for the HE. What is more, the 8° plantar flexion on the sagittal view and the secondary osteoarthritis of the ankle were observed (Fig. 2D).

The TKA (Legion, Smith & Nephew, Memphis, TN) was performed through a new anterior longitudinal incision and a medial parapatellar approach. The patella eversion was relatively easy because there were no apparent patella baja or patellar tendon shortening preoperatively. The cartilage on the front part of the tibial plateau was seriously worn. The cruciate ligaments, hyperplastic osteophyte, or soft tissue were cleared properly for the following resection. The distal femoral resection and the placement of the femoral component were performed normally as in a primary TKA. Given the bone stock loss of the proximal tibia resulting from the previous lateral closing wedge HTO, we minimized the amount of bone resection at the tibial plateau. To rebuild the posterior slope of the tibia, we increased the slope of the tibial resection and the thickness of the resection at the posterior tibial plateau was larger. The tibial component was placed slightly more medial to prevent impingement of tibial stem on the lateral cortex. A moderate degree of lateral laxity in varus stress was seen after the resection and medial release was adopted to balance the ligaments. A 13 mm polyethylene insert was used to reconstruct the JLH and the coronal ligament tension. The varus and HE deformity was corrected and the range of motion after suture was 0 to 160°.

**Figure 3:**
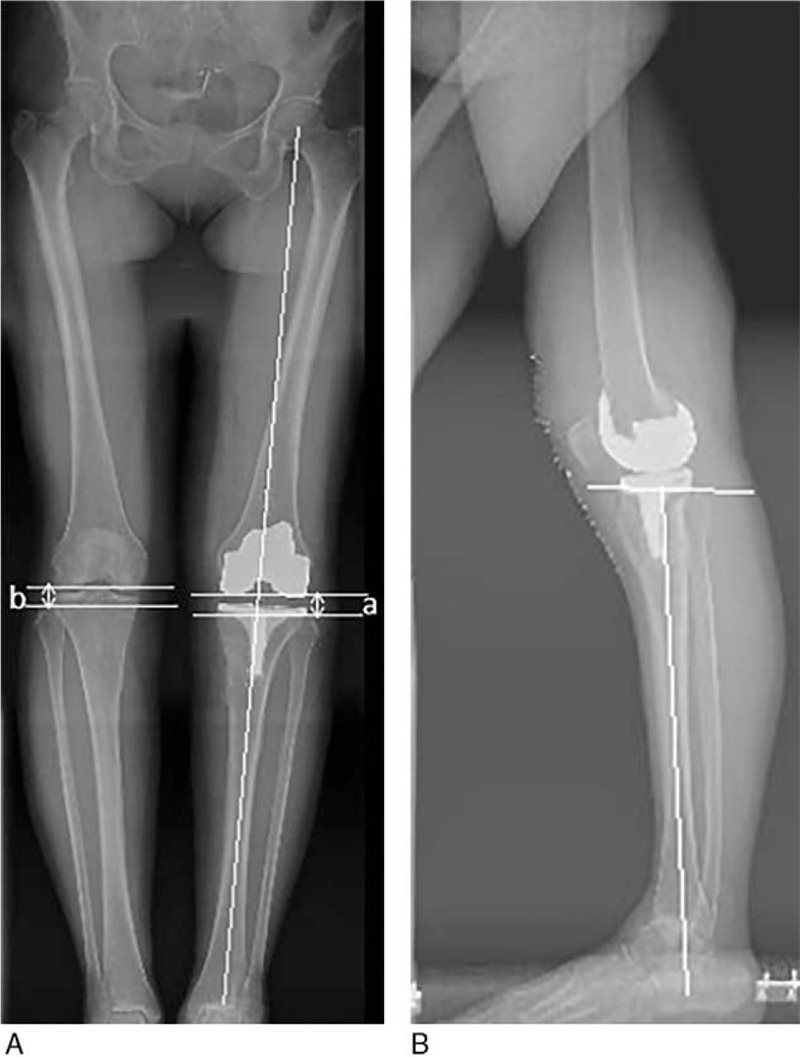
The postoperative weight-bearing x-ray films. (a) The coronal weight-bearing x-ray showed neutral alignment. The JLH was reconstructed by minimizing the amount of the tibial resection and using a thick polyethylene insert. (b) The sagittal x-ray showed the postoperative aPPTA was 86°. The deformity of HE and posterior subluxation of the tibia were corrected. aPPTA = anatomic posterior proximal tibial angle, HE = hyperextension, JLH = joint line height.

**Figure 4:**
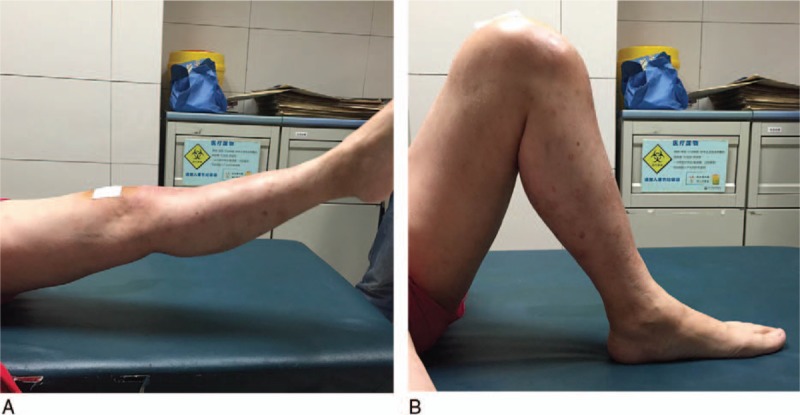
(A and B) The active range of motion was 0 to 135° 2 months after the operation.

The postoperative weight-bearing x-ray (Fig. 3A and B) showed favorable limb alignment on both the coronal and sagittal views. The aPPTA was 86° and the deformity of HE and posterior subluxation of the tibia were corrected. Two months later, there was no pain at her left knee and she could walk unaided. Active range of motion was 0 to 135° (Fig. 4A and B). The HSS score of the left knee was 85 three month after the surgery. However, the swelling and pain at the level of the ankle were still apparent. The deformity and osteoarthritis of the left ankle could not be reversed completely.

## 3 Discussion

Although HTO has been regarded as a good treatment for varus knee osteoarthritis for decades, conversion to TKA is still needed in some patients shortly after an HTO. Compared with medial opening wedge HTO, the disadvantages of lateral closing wedge HTO include relatively complex surgery procedures, loss of the bone stock, possible complications, and increased technical difficulty in the following TKA.^[9]^ In this case report, we discussed the radiographic and clinical outcomes of a lateral closing wedge HTO and its influence on the following TKA.

Excessive decrease of the tibial posterior slope mainly accounted for the failure of the HTO in this case. The tibia has a triangular shape with the apex directed anteriorly, so osteotomy may result in changes not only on the coronal plane, but also on the sagittal plane. The osteotomy causes more bone loss anteriorly and subsequent compression results in a reduction of the slope.^[11]^ The change of the tibial slope may have an influence on the kinematics and stability of the knee.^[12]^ Previous study has reported that decreasing the slope causes posterior translation of the tibia and overload on the posterior cruciate ligament (PCL) simultaneously. The posterior stability and the range of the knee are reduced consequently.^[13]^ In addition, the HE of the knee due to the slope reduction increases the load of the anterior part of the tibial plateau, which might speed up osteoarthritis progression. What is more, the secondary deformity and osteoarthritis of the ankle could not be ignored in this patient and this can be explained by the compensatory mechanism of the gait. To obtain a plant grade foot, the plantar flexion compensated for the HE of the knee. The ankle contact area decreases and moved posteriorly.^[14,15]^ The higher contact pressure between the distal tibia and talus results in secondary degeneration and pain of the ankle.

**Figure 5:**
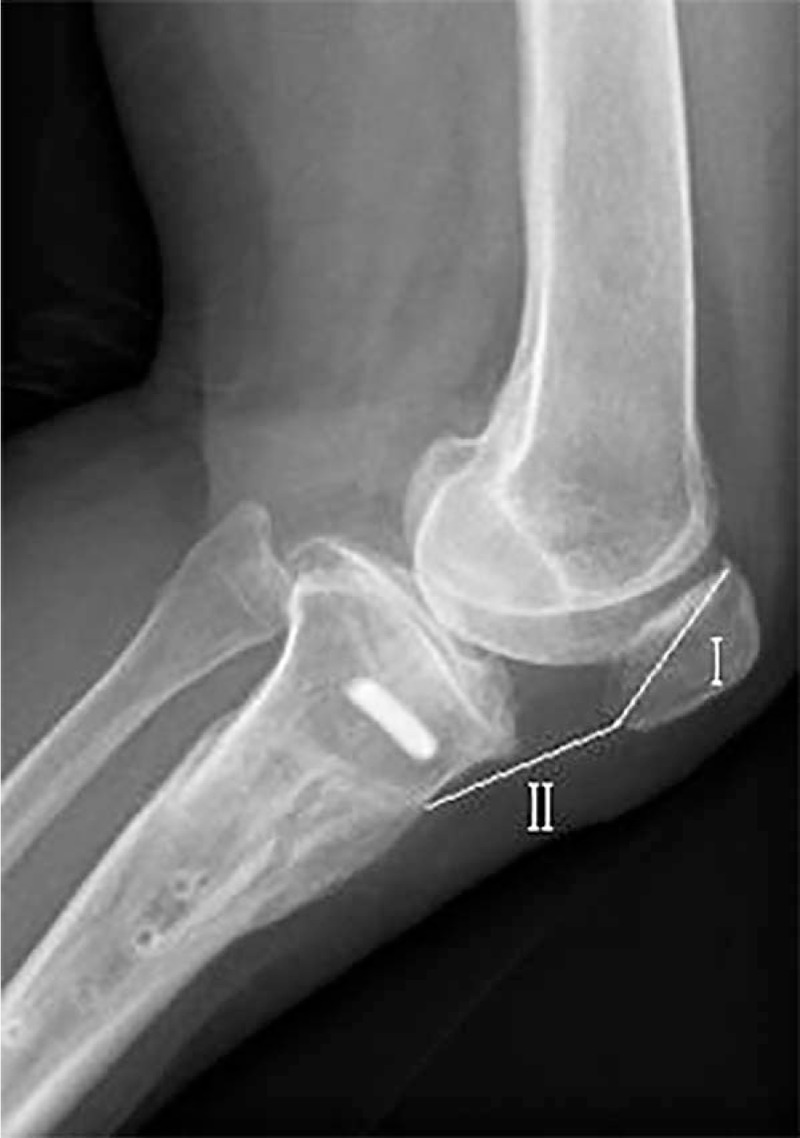
The biplanar osteotomy with osteotomy preserves the distance from the tuberosity to the joint. The Insall–Salvati ratio (II/I) was 1.08 and changed little after HTO. HTO = high tibial osteotomy.

For conventional Coventry closing wedge osteotomy, the change of the patellar height is another factor affecting the results of the HTO and the following TKA. The site of the osteotomy is proximal to the tibial tuberosity and the tuberosity moves closer to the knee joint line. The patella may ride proximally because of the retinaculum, creating a pseudo-patella alta.^[16]^ In another situation when the knee is splinted, the patellar is kept in positon. It could cause tendon scaring and pseudo-patella baja ^[17]^ and make the surgery exposure of the TKA difficult. However, the patient in this case underwent a biplanar lateral closing wedge osteotomy with osteotomy exits distal to the tibial tuberosity. The Insall-Salvati ratio was 1.08 and changed little after HTO (Fig. 5). The distance from the tuberosity to the joint was preserved and the problems about patellar height was minimized.^[18]^

In addition to the change of the tibial slope and patellar height, the lower limb shortening, JHL reduction and lateral ligament resection laxity are also intrinsic characteristics of lateral closing wedge HTO.^[7–9]^ Previous studies have compared the TKA following a failed lateral closing wedge HTO and the standard primary TKA on the postoperative radiographic and clinical outcomes with inconsistent conclusions. Some authors reported no substantially difference in clinical outcomes between these 2 groups although a previous closing wedge HTO may cause some important changes including malalignment, patella baja, bone stock loss, and the morphological change of the proximal tibia,^[19,20]^ whereas the others advocated the opposite views and reported inferior results in those with a previous HTO.^[21,22]^ However, it is recognized that there will be increasing technical complexity in converting a closing wedge HTO to a TKA than a primary TKA. The patellar baja and patellar tendon shortening make the surgery exposure difficult. Some cases need quadriceps snip and tibial tuberosity osteotomy.^[9]^ The tibial bone stock loss increases the risk of impingement between the stem of the tibial tray and the lateral cortex. The surgeon need to use undersized tibial trays, leave the lateral tibial plateau uncovered partially or put the tibial tray medially to solve the problem.^[23]^ In addition, extra operation time is spared to remove the residual hardware, reconstruct the JLH, adjust the tibial posterior slope, and balance the ligaments.^[24]^

## 4 Conclusion

The favorable outcomes of a lateral closing wedge HTO rely on reasonable patient selection, careful preoperative planning, and exquisite surgical techniques. A failed HTO could result in unexpected knee malalignment on the sagittal plane and accelerate the progression of the knee osteoarthritis. The secondary deformity and degeneration of the ankle can neither be ignored. What is more, extra technical issues should be considered in converting a failed lateral closing wedge to TKA.

## References

[1] Coventry MB Osteotomy of the upper portion of the tibia for degenerative arthritis of the knee. A preliminary report. J Bone Joint Surg Am 1965;47:984–90. 14318636

[2] A WD Toksvig-Larsen S Lindstrand A Ten-year results of physical activity after high tibial osteotomy in patients with knee osteoarthritis. Knee Surg Sports Traumatol Arthrosc 2017;25:902–9. 26170187 10.1007/s00167-015-3693-6

[3] Cerciello S Vasso M Maffulli N Total knee arthroplasty after high tibial osteotomy. Orthopedics 2014;37:191–8. 24762146 10.3928/01477447-20140225-08

[4] Naudie D Bourne RB Rorabeck CH The Install Award. Survivorship of the high tibial valgus osteotomy. A 10- to 22-year followup study. Clin Orthop Rel Res 1999 18–27. 10546594

[5] Insall JN Hood RW Flawn LB The total condylar knee prosthesis in gonarthrosis. A five to nine-year follow-up of the first one hundred consecutive replacements. J Bone Joint Surg Am 1983;65:619–28. 6853567

[6] Amendola A Unicompartmental osteoarthritis in the active patient: the role of high tibial osteotomy. Arthroscopy 2003;19 (suppl 1):109–16. 14673428 10.1016/j.arthro.2003.09.048

[7] Han JH Yang JH Bhandare NN Total knee arthroplasty after failed high tibial osteotomy: a systematic review of open versus closed wedge osteotomy. Knee Surg Sports Traumatol Arthrosc 2016;24:2567–77. 26423055 10.1007/s00167-015-3807-1

[8] Lee DC Byun SJ High tibial osteotomy. Knee Surg Rel Res 2012;24:61–9. 10.5792/ksrr.2012.24.2.61 PMC3374001 22708105

[9] Bastos Filho R Magnussen RA Duthon V Total knee arthroplasty after high tibial osteotomy: a comparison of opening and closing wedge osteotomy. Int Orthop 2013;37:427–31. 23288047 10.1007/s00264-012-1765-5 PMC3580093

[10] Paley D Principles of Deformity Correction. Berlin: Springer; 2002.

[11] Hohmann E Bryant A Imhoff AB The effect of closed wedge high tibial osteotomy on tibial slope: a radiographic study. Knee Surg Sports Traumatol Arthrosc 2006;14:454–9. 16292683 10.1007/s00167-005-0700-3

[12] Giffin JR Vogrin TM Zantop T Effects of increasing tibial slope on the biomechanics of the knee. Am J Sports Med 2004;32:376–82. 14977661 10.1177/0363546503258880

[13] Petrigliano FA Suero EM Voos JE The effect of proximal tibial slope on dynamic stability testing of the posterior cruciate ligament- and posterolateral corner-deficient knee. Am J Sports Med 2012;40:1322–8. 22427622 10.1177/0363546512439180

[14] Wagner KS Tarr RR Resnick C The effect of simulated tibial deformities on the ankle joint during the gait cycle. Foot Ankle 1984;5:131–41. 6519604 10.1177/107110078400500306

[15] Leung J Smith R Harvey LA The impact of simulated ankle plantarflexion contracture on the knee joint during stance phase of gait: a within-subject study. Clin Biomech (Bristol, Avon) 2014;29:423–8. 10.1016/j.clinbiomech.2014.01.009 24529471

[16] Tigani D Ferrari D Trentani P Patellar height after high tibial osteotomy. Int Orthop 2001;24:331–4. 11294424 10.1007/s002640000173 PMC3619923

[17] Westrich GH Peters LE Haas SB Patella height after high tibial osteotomy with internal fixation and early motion. Clin Orthop Rel Res 1998 169–74. 10.1097/00003086-199809000-00020 9755776

[18] Murphy SB Tibial osteotomy for genu varum. Indications, preoperative planning, and technique. Orthop Clin N Am 1994;25:477–82. 8028888

[19] Meding JB Keating EM Ritter MA Total knee arthroplasty after high tibial osteotomy. A comparison study in patients who had bilateral total knee replacement. J Bone Joint Surg Am 2000;82:1252–9. 11005516 10.2106/00004623-200009000-00005

[20] Haddad FS Bentley G Total knee arthroplasty after high tibial osteotomy: a medium-term review. J Arthroplasty 2000;15:597–603. 10959998 10.1054/arth.2000.6621

[21] Parvizi J Hanssen AD Spangehl MJ Total knee arthroplasty following proximal tibial osteotomy: risk factors for failure. J Bone Joint Surg Am 2004;86-A:474–9. 14996871 10.2106/00004623-200403000-00003

[22] Haslam P Armstrong M Geutjens G Total knee arthroplasty after failed high tibial osteotomy long-term follow-up of matched groups. J Arthroplasty 2007;22:245–50. 17275642 10.1016/j.arth.2006.01.031

[23] Kazakos KJ Chatzipapas C Verettas D Mid-term results of total knee arthroplasty after high tibial osteotomy. Arch Orthop Trauma Surg 2008;128:167–73. 18008079 10.1007/s00402-007-0488-3

[24] Gupta H Dahiya V Vasdev A Outcomes of total knee arthroplasty following high tibial osteotomy. Indian J Orthop 2013;47:469–73. 24133306 10.4103/0019-5413.118202 PMC3796919

